# Transferrin Receptor Overexpression in Solid Tumors Is Associated with Inflamed Microenvironments and Upregulated Immune Checkpoints, with Implications for Immunotherapy Sensitivity

**DOI:** 10.3390/cancers18091402

**Published:** 2026-04-28

**Authors:** Asaad Trabolsi, Marianna Lekakis, Peter M. Commisso, Nishant Gandhi, Andrew Elliott, Stephen V. Liu, Patrick C. Ma, Dave S. B. Hoon, Shuanzeng Wei, Emmanuel S. Antonarakis, Artavazd Arumov, Jonathan H. Schatz

**Affiliations:** 1 Sylvester Comprehensive Cancer Center, University of Miami Miller School of Medicine, Miami, FL 33136, USA; axt312@med.miami.edu (A.T.); mxl1628@miami.edu (M.L.); aarumov@miami.edu (A.A.); 2 Division of Hematology, Department of Medicine, University of Miami Miller School of Medicine, Miami, FL 33136, USA; 3 University of Miami, Coral Gables, FL 33124, USA; pmc132@miami.edu; 4 Caris Life Sciences, Phoenix, AZ 85040, USA; ngandhi@carisls.com (N.G.); aelliott@carisls.com (A.E.); 5 Lombardi Comprehensive Cancer Center, Georgetown University, Washington, DC 20007, USA; stephen.liu@gunet.georgetown.edu; 6 LSU-LCMC Health Cancer Center, Louisiana State University Health Science Center, New Orlans, LA 70112, USA; pma@lsuhsc.edu; 7 St. John’s Cancer Institute, Providence Healthcare Services, Santa Monica, CA 90404, USA; dave.hoon@providence.org; 8 Fox Chase Cancer Center, Philadelphia, PA 19111, USA; shuanzeng.wei@fccc.edu; 9 Masonic Cancer Center, University of Minnesota-Twin Cities, Minneapolis, MN 55455, USA; anton401@umn.edu

**Keywords:** transferrin receptor, immune evasion, ferroptosis, TFR1, *TFRC*

## Abstract

**Background/Objectives:** Overexpression of transferrin receptor (TFR1) is common in cancer and may be associated with inferior treatment outcomes. Due to these patterns and TFR1’s essential role in iron metabolism, the protein has been targeted for cytotoxic drug delivery. More recently, increased TFR1 expression has been linked to tumor microenvironment (TME) infiltration by immune effectors in selected tumors, but a comprehensive assessment of the genomic landscape associated *TFRC* (the gene encoding TFR1) expression has not been conducted. **Methods:** By utilizing a pan-cancer database of 93,248 patients with whole-exome and whole-transcriptome sequencing, we assessed *TFRC*-associated multiomic patterns. **Results:** We found that high *TFRC* expression correlates with significantly worse overall survival in multiple common solid tumor types, a higher tumor mutational burden (TMB), an increase in infiltrating effector cells with upregulated immune checkpoint markers within the TME, and increased frequency of specific high-risk genomic alterations. Further assessment in cell line models revealed increased susceptibility to cytotoxic T cells when iron metabolism is elevated, despite upregulation of the checkpoint ligand PD-L1. **Conclusions:** High *TFRC* expression, therefore, indicates worse clinical risk across multiple common tumor types but potentially increased susceptibility to cytotoxic immune effectors, informing the development of TFR1 biomarker-driven therapeutic strategies.

## Simple Summary

Transferrin receptor (TFR1) imports iron into cells and is often overexpressed in tumors. We assessed the prognostic impact of TFR1 expression in a curated collection of tumor samples from tens of thousands of cancer cases. We found that high expression is associated with worse overall survival in breast, prostate, and lung cancers and multiple other solid-tumor malignancies. Moreover, TFR1 is associated with common high-risk genomic tumor drivers. Assessment of gene expression patterns in tumor microenvironments (TMEs) showed a strong association between high TFR1 expression and infiltration by immune effector cells with high expression of clinically targetable immune checkpoint molecules. Treatment response data showed that immunotherapies directed against these molecules largely overcame the poor prognosis associated with high TFR1. Therefore, high TFR1 expression is a marker of worse overall survival in multiple common tumor types, but clinically available immune checkpoint therapies may have the potential to overcome its prognostic impact.

## 1. Introduction

Contemporary immuno-oncology (IO) has prompted a paradigm shift in assessments of tumor biology in relation to the immune system [1]. Over the past decade, cancer immunology research has successfully translated novel therapies into the clinic, with multiple FDA-approved agents, including immune checkpoint inhibitors (ICIs), monoclonal antibodies (Ab) targeting PD1, PD-L1, and CTLA4 [2,3]; adoptive T-cell transfer (ACT) therapy targeting CD19 and BCMA [4]; Ab-based T-cell engagers (TCEs) redirecting CD3+ T-cells against CD19, CD20, and BCMA [5]; and viral oncolytic therapy [6]. These advances have led to the first tumor-agnostic FDA approval in 2017 of the ICI pembrolizumab for tumors exhibiting high microsatellite instability (MSI-H) or mismatch repair deficiency (dMMR) [7]. While initial successes of ACTs and TCEs were largely in hematologic malignancies, the combination of IO and ACT research, as well as improved Ab engineering strategies, has resulted in FDA-approved IO medicines for use in solid tumors, including in metastatic melanoma (lifileucel) [8], uveal melanoma (tebentafusep-tebn) [9], and accelerated approval in small cell lung cancer (tarlatamab-dlle) [10]. Many challenges persist, however, in advancing more IO therapies for use across solid tumors [11,12], in particular, immune-repressive tumor microenvironments (TMEs) [13]. Monotherapy and combination regimens have been proposed as solutions [14,15], although with limited success to date. These challenges warrant further discovery and study of new IO therapeutic targeting strategies to provide new effective options for patients.

CD71, also known as transferrin receptor 1 (TFR1), encoded by *TFRC,* is a cell-membrane protein involved in the uptake and regulation of cellular iron [16,17]. Upon binding of iron-loaded transferrin (Holo-TF), TFR1 undergoes clathrin-mediated endocytosis and is trafficked to low-pH endosomes, where iron is released and used by the cell to fuel cellular growth, while TFR1 is recycled back to the cell surface. In non-malignant tissues, *TFRC* is generally expressed at the highest levels in rapidly proliferating progenitor populations. Cancer cells frequently reprogram iron metabolic machinery to support tumorigenesis and proliferation [18,19], and indeed, *TFRC* has been found to be overexpressed across a range of hematologic and solid tumors [20]. At times, however, tumors with high iron levels may specifically downregulate TFR1 to provide protection from ferroptosis [21,22,23]. Therefore, a unified analysis across multiple tumor types is warranted to reveal those malignancies in which upregulation confers the most consistent prognostic impact.

We previously showed that high *TFRC* is associated with a more aggressive diffuse large B-cell lymphoma (DLBCL) phenotype [24], and now we extrapolate these findings in a clinically focused manner across a range of solid tumor subtypes. We find that high *TFRC* expression correlates with a poorer prognosis for solid tumor disease [25], a finding previously reported in isolated disease-specific instances [26,27]. Given its role promoting tumorigenesis and its high on-tumor low off-tumor tissue expression, TFR1 has been proposed as a high-value anti-cancer therapy drug target, though attempts at targeting it so far have not progressed to regulatory approval [28,29]. We find *TFRC* to be implicated in prominent roles within the TME, with high expression correlating with increased immune-effector infiltration [30]. TFR1 is also a known marker of susceptibility to ferroptosis, a mechanism of cell death mediated by iron imbalance, leading to the accumulation of oxidized polyunsaturated fatty-acid-containing phospholipids [31,32]. While ferroptosis is linked to immunomodulatory effects in the TME [33] and contributes to the response to anti-PD-L1 Ab therapies [34] *TFRC* expression has not been assessed in a pan-cancer fashion. Thus, we sought to explore the genomic interplay between *TFRC*, the immune system, genomic disease drivers, and markers associated with ferroptosis across solid tumors, utilizing a real-world cancer cohort of 73,213 patients. We reveal increased tumor expression as a biomarker for microenvironments infiltrated by T cells expressing therapeutically targetable checkpoint markers. Moving forward, further elucidation of the role *TFRC* plays within the TME could inform the use of both current IO therapies and a variety of TFR1-directed therapies under development.

## 2. Materials and Methods

### 2.1. Patient Cohort

For genomic and immune analysis, specimens from non-small cell lung cancer (NSCLC: *N* = 42,326), colorectal cancer (CRC: *N* = 14,287), breast cancer (*N* = 10,728), and prostate adenocarcinoma (PRAD: *N* = 5872) were molecularly profiled at Caris Life Sciences (Phoenix, AZ, USA). Samples sent as part of the standard of care for next-generation sequencing (NGS) were included in the study. The data included biopsies obtained from primary and metastatic sites.

### 2.2. Next-Generation Sequencing of DNA

NGS was performed on genomic DNA isolated from formalin-fixed paraffin-embedded (FFPE) tumor samples using the NextSeq 550 or NovaSeq 6000 platform (Illumina, Inc., San Diego, CA, USA). For the Nextseq-sequenced tumors, a custom-designed SureSelect XT assay was used to enrich 592 whole-gene targets (Agilent Technologies, Santa Clara, CA, USA). Further, for the NovaSeq-sequenced tumors, 719 clinically relevant genes were enriched at high coverage and read depth, along with another panel designed to enrich for >20,000 genes at lower read depth. All variants were detected with >99% confidence based on allele frequency and amplicon coverage, with an average sequencing depth of coverage of >500 and an analytic sensitivity to identify variants with a variant allele frequency of ≥5%. Genetic variants identified were interpreted by board-certified molecular geneticists and categorized as ‘pathogenic (P)’, ‘likely pathogenic (LP)’, ‘variant of unknown significance’, ‘likely benign’, or ‘benign’, according to the American College of Medical Genetics and Genomics (ACMG) standards [35]. Only ‘pathogenic’ and ‘likely pathogenic’ variants were utilized to estimate mutation frequencies of individual genes. The copy number alteration (CNA) of each exon was determined by calculating the average depth of the sample along with the sequencing depth of each exon and comparing this calculated result to a pre-calibrated value.

### 2.3. Tumor Mutational Burden Estimation

After excluding all variants previously described as germline alterations according to dbSNP151, Genome Aggregation Database (gnomAD) databases, and those characterized as benign variants by Caris geneticists, tumor mutational burden (TMB) was calculated by counting non-synonymous, missense, nonsense, in-frame insertion/deletion, and frameshift mutations present in each tumor as previously described [36,37]. A cutoff point of ≥10 mutations per MB was used to classify TMB-high (TMB-H) tumors, based on the findings from the KEYNOTE-158 study [38,39].

### 2.4. RNA Sequencing

Gene fusion detection was performed on mRNA isolated from a formalin-fixed paraffin-embedded tumor sample using the Illumina NovaSeq platform (Illumina, Inc., San Diego, CA, USA) and Agilent SureSelect Human All Exon V7 bait panel (Agilent Technologies, Santa Clara, CA, USA). FFPE specimens underwent pathology review to measure tumor content and tumor size; a minimum of 10% tumor content in the area for microdissection was required to enable enrichment and extraction of tumor-specific RNA. The Qiagen RNA FFPE tissue extraction kit was used for RNA extraction, and the RNA quality and quantity were determined using the Agilent TapeStation. Biotinylated RNA baits were hybridized to the synthesized and purified cDNA targets, and the bait-target complexes were amplified in a post-capture PCR reaction. The resultant libraries were quantified, normalized, and the pooled libraries were denatured, diluted, and sequenced; the reference genome used was GRCh37/hg19 and analytical validation of this test demonstrated ≥97% Positive Percent Agreement (PPA), ≥99% Negative Percent Agreement (NPA) and ≥99% Overall Percent Agreement (OPA) with a validated comparator method.

For RNA expression, the whole transcriptome from tumor samples was sequenced to an average of 60 M reads. Raw data were demultiplexed by the Dragen BioIT accelerator (Illumina Inc., San Diego, CA, USA), trimmed, counted, PCR duplicates were excluded, and aligned to the human reference genome hg19 by the STAR aligner. TPM (Transcripts Per Million Molecules) were generated using the Salmon expression pipeline [40].

Cells comprising the tumor microenvironment were estimated using the quanTIseq immune deconvolution method from bulk RNA sequencing [41].

### 2.5. Immunohistochemistry (IHC)

IHC for Programmed Death-Ligand 1 (PD-L1) was performed using the 22c3 Ab (Dako City) in accordance with the manufacturer’s protocol and was further optimized and validated per CLIA/CAO and ISO requirements. Tumor Proportion Score (TPS) was measured as the percentage of viable tumor cells showing partial or complete membrane staining at any intensity. PD-L1+ was defined as a TPS ≥ 1%.

### 2.6. Survival Analysis

Real-world overall survival (OS) information was obtained from insurance claims data and calculated from the time of tissue biopsy to the last contact. Unless labeled as IO-OS, this cohort did not include patients treated with IO. Survival on any IO (IO-OS) was calculated from the initiation of pembrolizumab, nivolumab, atezolizumab, durvalumab, or ipilimumab to the last contact to avoid confounding of prior lines administered. Hazard ratios (HR) were calculated using the Cox proportional hazards model, and *p* values were calculated using the log-rank test.

### 2.7. Statistics

Chi-square, Fisher’s Exact test, Mann–Whitney U test, and *t*-tests (with Welch’s correction) were used and adjusted for multiple hypothesis testing using the Benjamini–Hochberg method where appropriate. Statistical significance was considered when false discovery rate q < 0.05.

### 2.8. Cell Culture and Treatments

A-549, NCI-H460, and NCI-H2228 cell lines were kindly provided by Dr. Priyamvada Rai (U. Miami) and underwent mycoplasma testing and identity confirmation by STR fingerprinting prior to use. They were maintained in Dulbecco’s Modified Eagle Medium (DMEM) supplemented with 10% fetal bovine serum (FBS), 5 μg/mL Plasmocin (InvivoGen), 100 IU/mL Penicillin, and 100 mg/mL Streptomycin (Gibco, Thermo Fisher Scientific, Waltham, MA, USA). NCI-H358 was maintained in RPMI supplemented with 10% FBS, 5 μg/mL Plasmocin, 100 IU/mL Penicillin, and 100 mg/mL Streptomycin. Each line was cultured in an incubator at 37 °C with 5% CO_2_, verified by the STR fingerprinting assay kit, and regularly tested for mycoplasma.

For treatments, three million NCI-H460 and NCI-H358 cells were plated 24 h before the start of the experiment. The following day, cells were treated with 10 µM or 50 µM Deferoxamine (DFO) (Selleck Chemicals LLC, Houston, TX, USA, S5742) for 24 h with DMSO alone as a control, or with 100 µM, 500 µM, or 1 mM of Ferric Ammonium Citrate (FAC) (MilliporeSigma, Burlington, MA, USA, F5879-100G) with water as a control. At the end of treatments, protein was extracted for Western blot analysis.

### 2.9. Co-Culture

Cytotoxic (CD8) T-cells were isolated from pooled-donor peripheral blood mononuclear cells (PBMCs) (StemCell Technologies, Vancouver, BC, Canada, 70025.3) using the EasySep™ Human CD8+ T Cell Isolation Kit (StemCell Technologies, Vancouver, BC, Canada, 17953) and maintained in RPMI media supplemented with 10% FBS, 5 μg/mL Plasmocin, 100 IU/mL Penicillin, and 100 mg/mL Streptomycin. The CD8 T-cells were cultured for 24 h before stimulation. For stimulation, 80 ng/mL of IL-2 (Thermo Fisher Scientific, Waltham, MA, USA, PHC0023) and a 1:1 bead-to-cell ratio of anti-CD3/anti-CD28 beads (ThermoFisher Scientific, 11131D) were added to the media to stimulate the cells overnight.

For co-culture, 2 million NCI-H460 cells were plated, and 24 h later, cells were treated with either 1 mM FAC or 50 µM DFO along with DMSO as a control. Twenty-four hours post-treatment, cells were washed and re-plated in RTCA E-Plates (Agilent) according to the manufacturer’s protocol. The following day, the stimulated CD8 T-cells were added after magnetic bead removal. The ratio of CD8 T-cells to H-460 cells was 3:1. The plate was placed on xCELLigence (Agilent Technologies, Santa Clara, CA, USA), and cell adherence was assessed over a period of 50 h.

### 2.10. Cell-Viability Assays

For the cell viability assay, 96-well white, flat-bottom plates were used, and cells (5000/well) were plated 24 h before the start of the experiments in two sets. The following day, cells were treated for 72 h with different concentrations of doxorubicin (MedChem Express, Monmouth Junction, NJ, USA, HY-15142), ranging from 5 μM to 9.77 nM and NanoDox (synthesized by Dr. Roger LeBlanc’s lab, UM Dept. of Chemistry [24]), serially diluted from 1 μM to 1.96 nM. Post-treatment, CellTiter-Glo (Promega, Madison, WI, USA, G7570) was added to the plates, and luminescence was measured using a plate reader. Nonlinear fit regression analysis was performed using GraphPad Prism v11.0.1 to determine EC_50_.

### 2.11. Western Blot Analysis

Whole-cell lysates were prepared using RIPA lysis buffer (ThermoFisher Scientific, J62725.AP) with 1X protease and phosphatase inhibitor cocktail. Cells were rotated at 4 °C for 30 min and then spun down at 1300 rpm for 10 min at 4 °C. The supernatant was collected, and the protein was quantified using the Bradford Assay (Bio-Rad, Hercules, CA, USA, 5000006).

Proteins were resolved on a 4–12% Bis-Tris pre-made gel (ThermoFisher Scientific) and transferred for one hour at 26 V to a 0.2 μm pore size Immuno-Blot PVDF Membrane (Bio-Rad), followed by blocking in 5% fat-free milk (Bio-Rad, 1706404) in TBS buffer (ThermoFisher Scientific) containing 0.5% Tween^®^ 20 detergent (Millipore Sigma). The membranes were cut into parts according to the molecular weight of the proteins of interest and probed with the primary antibodies diluted in blocking buffer overnight at 4 °C. Unbound antibodies were removed by washing three times for 10 min each with TBST buffer (TBS and 0.5% Tween^®^ 20). Membranes were then incubated in horseradish peroxidase (HRP) conjugated host-specific secondary antibodies for one hour. An enhanced chemiluminescent (ECL) substrate was used to detect the chemiluminescent signal using a digital chemiluminescent imaging system (LiCor).

The primary Ab used were: CD71 (D7G9X) XP^®^ Rabbit mAb (Cell Signaling, Danvers, MA, USA, 13113), PD-L1 (E1L3N) XP^®^ Rabbit mAb (Cell Signaling, 13684), β-Actin (8H10D10) Monoclonal mAb (Cell Signaling, 3700), and Cyclophilin B Polyclonal Antibody (ThermoFisher Scientific, PA1-027A). The secondary antibodies used were Anti-Rabbit IgG, HRP-linked Antibody (Cell Signaling, 7074), and Anti-Mouse IgG, HRP-linked Antibody (Cell Signaling, 7076). The whole blots (uncropped blots) are shown in Supplementary file.

## 3. Results

### 3.1. Pan-Cancer Analysis of TFRC Gene Expression

**Figure 1:**
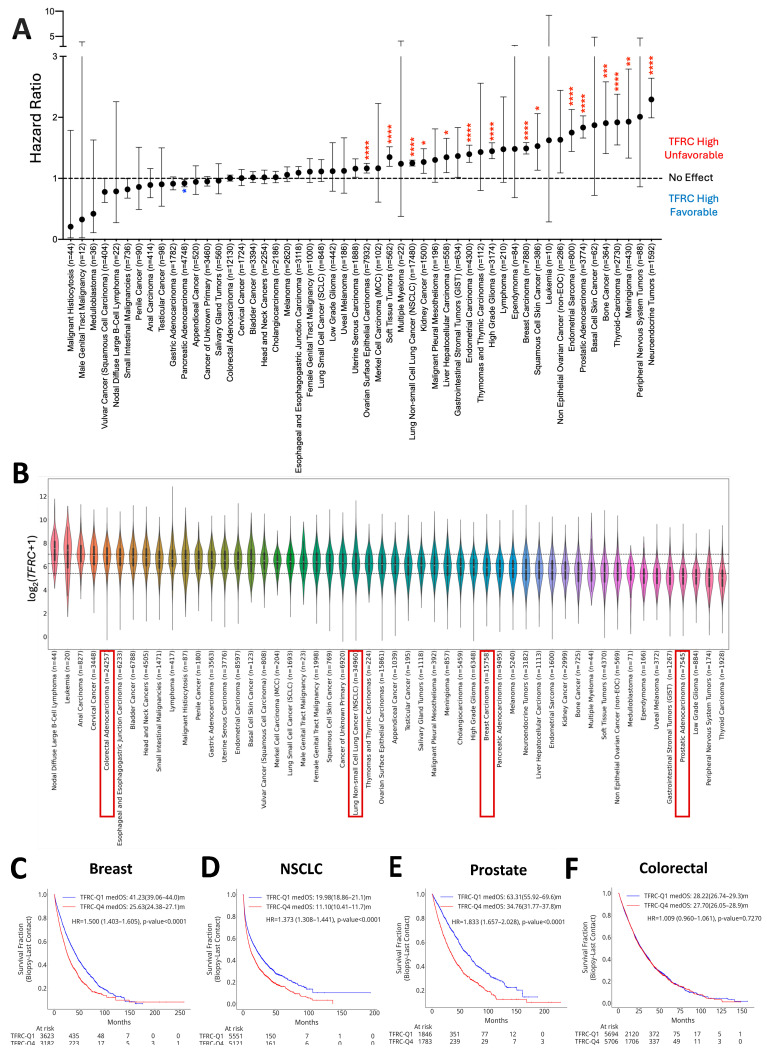
Pan-cancer prognostic analyses across tumor types. (**A**) OS was analyzed across a variety of tumors, each segregated into two groups (TFRC-high vs. TFRC-low). Asterisks denote tumor types for which high (red) or low (blue) *TFRC* expression was statistically significantly associated with OS. The most common solid tumors were stratified into high expressors (top quartile of *TFRC* expression-Q4) or low expressors (bottom quartile of TFRC expression-Q1) of *TFRC*. (**B**) Median mRNA expression assessed across tumors, with dashed lines representing the 25th, 50th, and 75th percentiles from top to bottom. The red box denotes the 4 most common solid tumor types. Kaplan–Meier estimates for OS were analyzed in (**C**) breast cancer, (**D**) non-small cell lung cancer, (**E**) prostate cancer, and (**F**) colorectal cancer. The Cox proportional hazard model was used to estimate the HR for each tumor type, with significance determined as *p* value of <0.05. * *p* < 0.05, ** *p* < 0.01, *** *p* < 0.001, **** *p* < 0.0001.

We undertook pan-cancer analysis of *TFRC* in 93,248 cases from 48 tumor types, segregating cases into quartiles by *TFRC* expression and comparing Q1 to Q4 (*TFRC*-low and *TFRC*-high, respectively) within each tumor type. Higher expression of *TFRC* was associated with a higher hazard ratio (HR) for death in 14 of 48 tumor types (*p* < 0.05 for all) (Figure 1A). Looking at median RNA expression of *TFRC* across solid tumors, we found colorectal cancer (CRC) and non-small cell lung cancer (NSCLC) above the 25th percentile, breast cancer at the 50th percentile, and PRAD at the 75th percentile (Figure 1B). We chose to further analyze the top 4 most common solid tumors, based on annual incidence (n = 73,213). Breast cancer (HR = 1.49, 95% CI 1.40–1.58) (Figure 1C), non-small cell lung cancer (NSCLC) (HR = 1.25, 95% CI 1.20–1.29) (Figure 1D), and PRAD (HR = 1.83, 95% CI 1.66–2.02) (Figure 1E) all demonstrated impaired overall survival (OS) in *TFRC*-high compared to *TFRC*-low cases, while there was no significant difference for CRC (HR = 1.009) (Figure 1F). *TFRC*-high was associated with significantly shorter median OS when analyzing clinically relevant molecular subclassifications, including estrogen receptor-positive/human epidermal growth factor receptor 2-null BC (ER+/HER2- BC) (Supplemental Figure S1A), adeno NSCLC (Supplemental Figure S1B), and AR-V7+ PRAD (Supplemental Figure S1C). Stratification of microsatellite stable (MSS) and microsatellite instable (MSI) colorectal cancer (CRC) did not result in any significant OS differences among *TFRC*-high vs. *TFRC*-low subsets (Supplemental Figure S1D). Likewise, no significant differences in OS were observed across HER2+ or triple-negative BC (TNBC) (Supplemental Figure S1A) or squamous NSCLC (Supplemental Figure S1B). These results reveal the prognostic impact of *TFRC* in a majority of the most common tumors.

### 3.2. High TFRC Expression Is Associated with Alterations of Specific Genes Including MYC and TP53

**Figure 2:**
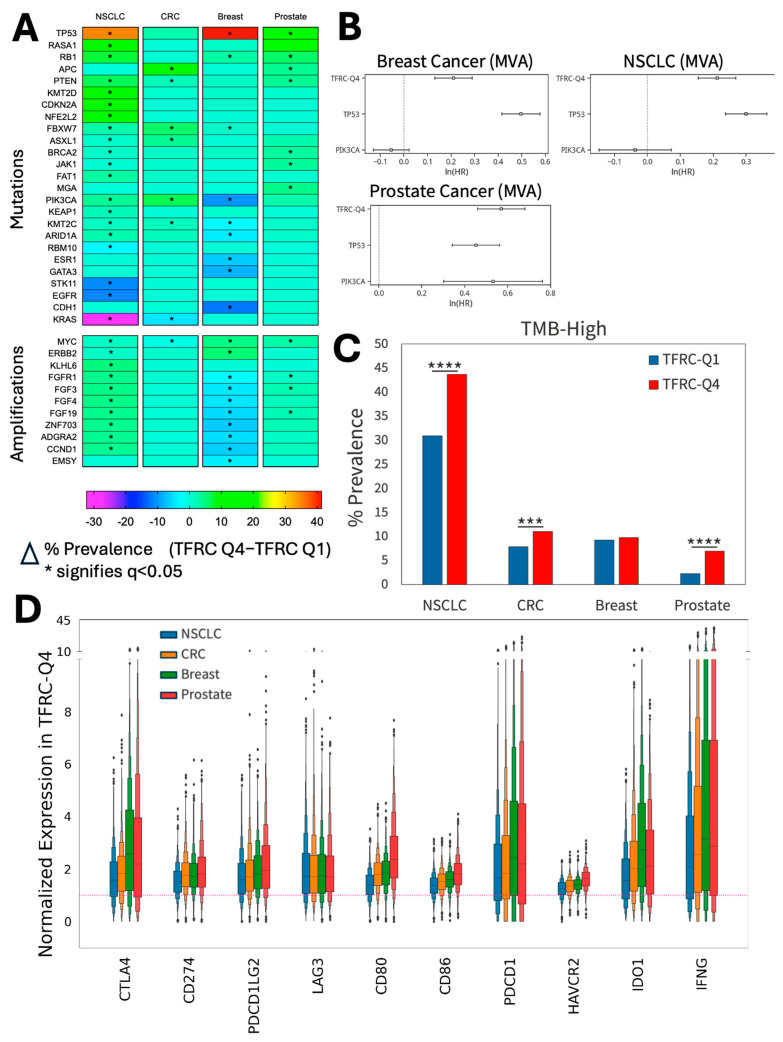
Association of *TFRC* expression with key tumors and immune drivers. (**A**) Prevalence of known tumor genomic amplifications compared between *TFRC*-high and low-expressing solid tumors. Genes with an asterisk depict statistical significance in the respective tumor type. (**B**) Multivariate analyses of breast, prostate, and lung cancers defining independent impact on OS of *TFRC*-high, *TP53* mutations, and *PIK3CA* mutations. See statistical tables, Supplementary Figure S2A. (**C**) Prevalence of TMB compared between *TFRC*-high and low tumor types. (**D**) Associations of common immune checkpoint and immune-regulatory proteins in *TFRC*-high tumors. *TFRC*-high is depicted as the upper quartile, *TFRC*-Q4. *TFRC*-low is depicted as the lower quartile, *TFRC*-Q1. Dotted red line is average expression for each tumor type, all cases. Statistical significance by false discovery rate = *, q < 0.05 (panel **A**). * *p* < 0.05, *** *p* < 0.001, **** *p* < 0.0001 (panel **C**).

The association between *TFRC* expression and various genomic alterations and biological pathways is largely unknown. Utilizing whole-exome and whole-transcriptome sequencing data from 73,213 samples of the most common solid tumor types (NSCLC, CRC, BC, PRAD), we found *TFRC*-high to be recurrently associated with *MYC* amplifications in each tumor type, with *TFRC*-high NSCLC harboring the most mutations in common oncogenic drivers (Figure 2A). We also found at least three *TFRC*-high tumor types to be recurrently associated with increased rates of pathogenic mutations in *TP53*, *RB1*, *PTEN*, *FBXW7*, *PIK3CA*, and *KMT2C*, as well as copy-number amplification of *FGFR1*, *FGF3*, and *FGF19* (Figure 2A). Given the association with drivers carrying potential negative prognostic impact, we next carried out multivariate analyses (MVA) with inclusion of *TFRC*-high, *TP53* mutations, and *PIK3CA* mutations (Figure 2B). In breast, prostate, and lung cancers, *TFRC*-high was independently associated with impaired overall survival, as were *TP53* mutations, while *PIK3CA* mutations were significant only in prostate cancer. Other gene alterations were too infrequent or inconsistent across the tumor types for inclusion in this analysis. Statistical tables for MVA analyses and *TFRC*-high OS curves with and without mutation of these genes are provided in Supplemental Figure S2A–D. Interestingly, we also found that *TFRC*-high NSCLC, MSS CRC, and PRAD had a positive association with an increased TMB (Figure 2C). Considering the association of mutated genes and TMB across the selected tumor types, we next probed immune checkpoint expression in *TFRC*-high vs. *TFRC*-low tumors. Consistent associations were found between *TFRC*-high and increased expression of the genes encoding common immune checkpoint proteins PD-L1, PD-1, LAG3, CTLA-4, and additional immune-regulatory molecules, including CD80, CD86, HAVCR2, IDO1, and IFN-γ (Figure 2D). Therefore, *TFRC*-high tumors are associated with high-risk genetic alterations and markers of immune checkpoint engagement.

### 3.3. TFRC-High Tumors Have Immune-Infiltrated Tumor Microenvironments

**Figure 3:**
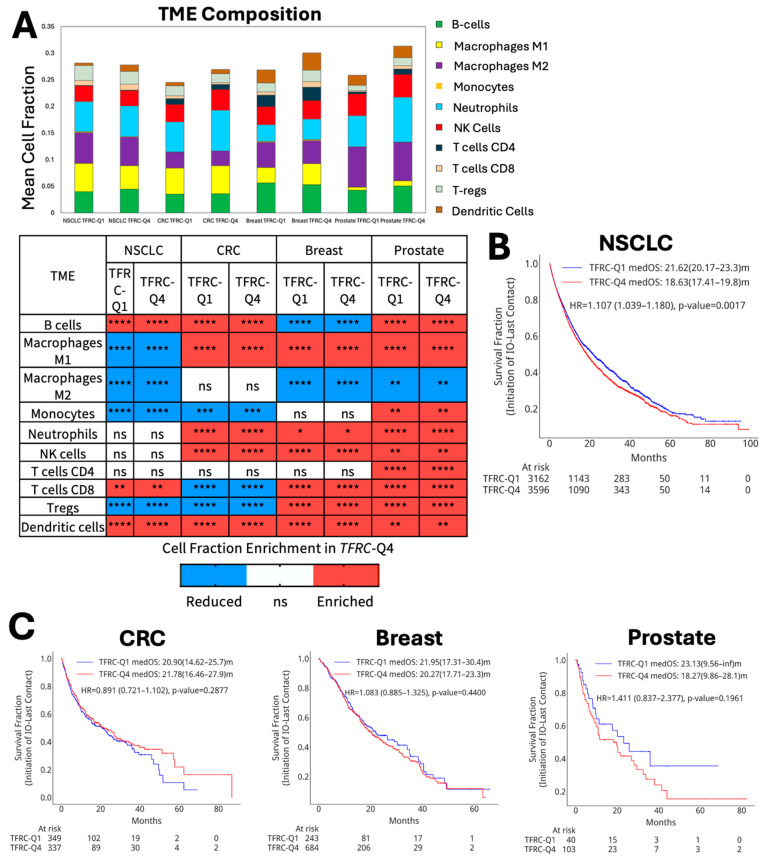
Impact of *TFRC* expression on the immune infiltrate and responsiveness to Immune checkpoint inhibitors. (**A**) Comparative immune cell infiltrate in *TFRC*-high and *TFRC*-low tumors (**top**), accompanied by quantified values (**bottom**). Red boxes represent a significant increase in immune infiltrate in *TFRC*-high tumors, and blue boxes represent a significant decrease in immune infiltrate in *TFRC*-high tumors. White boxes represent no significant difference. Kaplan–Meier estimates for OS analyzed in IO-treated *TFRC*-high and *TFRC*-low tumors across (**B**) NSCLC, (**C**) CRC, BC, and PRAD. Cox proportional hazards model was used to estimate the HR for each tumor type with significance determined as *p* value of <0.05. * *p* < 0.05, ** *p* < 0.01, *** *p* < 0.001, **** *p* < 0.0001.

Due to the expression of immune exhaustion markers in *TFRC*-high tumors, we next used immune deconvolution of bulk RNA expression profiles to infer TME subpopulation proportions. A general trend towards higher infiltration by immune cells was noted in *TFRC*-high tumors (Figure 3A). Dendritic cells were found at higher levels across all four tumor types, followed by consistently higher infiltration across at least three tumor types by B cells, neutrophils, NK cells, macrophages, and CD8 T cells (Figure 3A). Interestingly, we observed higher infiltration of anti-tumor M1 compared to M2 macrophages. We hypothesized that these TME alterations might impact tumor immunity and thus analyzed the survival on IO therapy in patients with *TFRC*-high tumors (Figure 3B,C). Among NSCLC patients with high *TFRC* expression, IO-OS remained worse compared to those with *TFRC*-low expressing tumors, but with a less pronounced hazard ratio (Figure 3B), with no observed differences in IO-OS across CRC, BC, and PRAD (Figure 3C). The otherwise negative prognostic impact of *TFRC* expression in these tumors, therefore, was ameliorated in NSCLC treated with ICI therapy and absent in other tumor types.

### 3.4. TFRC-High Tumors Overexpress Genes Associated with Protection from Ferroptosis

**Figure 4:**
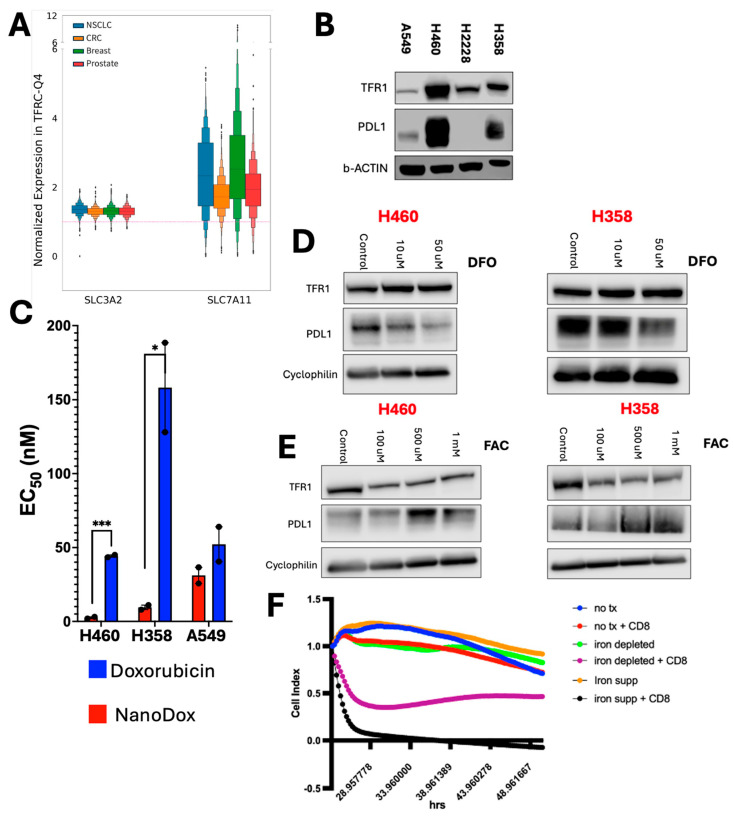
Impact of ferroptosis biology on tumor and immune protein expression. (**A**) Increased expression of ferroptosis genes in *TFRC*-high (upper quartile) tumors. Dotted red line is average expression for each tumor type, all cases. (**B**) Immunoblot assessment of TFR1 (CD71, 90 kD) and PD-L1 (CD274, 40 kD) with β-Actin loading control (42 kD) in untreated NSCLC cell lines A5949, H460, H2228, and H358. (**C**) EC_50_ values are defined as the concentration leading to 50% cell death after a 48 h tumor cell viability assay of H460, H358, and A549 cell lines plated in serial dilutions of doxorubicin or NanoDox. Immunoblot assessment of H460 and H358 cell lines treated with indicated concentrations of DFO (**D**) or FAC (**E**) for 24 h, with H_2_O as a control for both. (**F**) Tumor cell line H460 viability co-cultured with and without immune cells for 72 h in the presence or absence of iron supplementation and iron deprivation. * *p* < 0.05, *** *p* < 0.001.

Recent innovations in targeting TFR1 attempt to leverage TMEs for better anti-tumor activity, including conditionally activated Ab-drug conjugates targeting TFR1 [42], and a bispecific antibody that can deliver therapeutically relevant Ab past the blood–brain barrier, among others [43]. Induction of iron-mediated ferroptosis contributes to tumor cell killing by cytotoxic T cells, including responses induced by ICIs, which have been studied across solid tumors. Ferroptosis has also been implicated in driving inflammation within the TME [33,44,45]. Thus, we assessed ferroptosis markers relative to *TFRC*-associated TMEs in our solid tumor cohort. Strikingly, *TFRC*-high cases have significantly increased expression of solute carrier family 3 member 2 (*SLC3A2)* and *SLC7A11*, which together encode the System X_c_^−^ antiporter that fuels glutathione production, a key protective mechanism against ferroptosis [46] (Figure 4A).

Previous work assessing PD-L1 treatment revealed an induction of ferroptosis in murine models and downregulation of these same ferroptotic-protective genes, *SLC3A2* and *SLC7A11* [34]. These complementary sets of results led us to hypothesize that *TFRC*-high tumors require increased protection from ferroptosis, along with the induction of immune checkpoints, to avoid cytotoxic immune effectors in their inflamed TMEs. We therefore assessed whether manipulation of iron metabolism would impact PD-L1 expression and susceptibility to killing by cytotoxic T cells. Immunoblot assessment of a panel of *KRAS*-mutated NSCLC cell lines showed that two cell lines, H460 and H358, have concomitant high TFR1 and PD-L1 expression (Figure 4B). NanoDox is a nanoparticle-conjugated doxorubicin targeted to TFR1 through co-conjugation of holo-transferrin that we previously developed and tested in DLBCL [24]. Similar to those results, we found H460 and H358 are significantly more sensitive to NanoDox than unconjugated doxorubicin, while TFR1-low A549 showed no significant difference (Figure 4C). We next tested the effects of iron depletion and supplementation. Treatment with the iron-chelating agent deferoxamine (DFO) for 24 h caused both H460 and H358 to downregulate PD-L1 while TFR1 remained stable (Figure 4D). By contrast, iron supplementation with ferric ammonium citrate (FAC) increased PD-L1 expression in both lines (Figure 4E). Downregulation of TFR1 protein in response to iron supplementation is consistent with known mechanisms of protection from iron overload, including non-canonical endocytosis in which some TFR1 is degraded rather than recycled to the cell surface and induction of microRNAs that downregulate *TFRC* translation [47,48]. We next assessed the effect of co-culture with CD8 T cells, focusing on H460, which has constitutively activated NRF2 due to KEAP1 mutation and is resistant at baseline to both ferroptosis inducers and cytotoxic T cells [49]. We plated H460 on the xCELLigence Real-Time Cell Analysis system and allowed adherence to the electrode-imprinted plates in tissue culture for 24 h. Cells were untreated or exposed to DFO (50 µM) or FAC (1 mM) during adherence. We then replaced the media with fresh drug-free media with or without CD8 T cells purified from pooled-donor peripheral blood mononuclear cells (PBMCs). As expected, unpretreated H460 cells showed no significant response to the CD8 co-culture over 72 h (Figure 4F). H460 pretreated with FAC, however, rapidly died and had no remaining viability signal within a few hours after initiation of co-culture. Interestingly, iron depletion with DFO also increased the sensitivity of H460 to CD8 co-culture transiently. Neither iron manipulation alone significantly impacted the viability of the cells. These results are consistent with significantly increased sensitivity to cytotoxic immune effectors associated with more active iron metabolism, an effect that overcame the concomitant increase in expression of the PD-L1 immune checkpoint engager.

## 4. Discussion

TFR1 has been previously studied as a cancer therapeutic target on a tumor-by-tumor basis, as exemplified by reports of its high expression and negative prognostic impact. Fueled by advances in Ab engineering technology and following recent biological findings, several notable TFR1-targeting therapies have recently entered clinical trials. INA03 is an ADC consisting of a humanized anti-CD71 Ab with a monomethyl auristatin E (MMAE) payload [50] that is currently under clinical evaluation in a relapsed/refractory acute leukemia Phase 1 trial (NCT03957915). Preliminary data from the open-label Phase 1 trial showed a 30% response rate, with blast reductions observed in 6/20 patients, including two partial responses (PR) [51]. PROCLAIM-CX-2029 is an open-label Phase 1–2 trial assessing CX-2029 in advanced solid tumors and DLBCL (NCT03543813). CX-2029 is an anti-CD71 ADC with an MMAE payload, with a unique Ab engineering strategy utilized whereby the drug is conditionally activated to release the MMAE cytotoxic payload in the presence of a tumor-specific protease cleavage occurring within the TME [42]. Phase 1 findings led to the successful identification of a recommended Phase 2 dose of 3 mg/kg, with 2 of 10 evaluable patients achieving PRs (20%), and stable disease in another 4 (40%) [52]. CX-2029’s efficacy signal in the Phase 1 trial and its mechanism reliant on the TME tie in well with our study and should prompt further efforts to develop a deeper comprehension of TFR1 roles in tumor biology

In particular, we observed that a comprehensive assessment of clinicogenomic features of *TFRC* across solid tumors was lacking in the field. We therefore assessed genomic and transcriptomic alterations of tumors and their TME in disease types where high *TFRC* expression carried a negative prognostic impact. Expanding on the previous single-tumor-type findings [53,54,55], our data strengthen *TFRC*’s potential in clinical prognostication, specifically in BC, PRAD, and NSCLC, the three most common in the U.S. We find that *TFRC*-high tumors lead to significantly poorer OS, and are associated with driver alterations of known tumor-promoting genes, including *MYC*, *TP53*, *PIK3CA*, and *FGFR2*, as well as increased overall TMB (Figure 2A). Interestingly, some tumor types in which iron metabolism has been studied as a disease driver did not have worse OS associated with *TFRC*-high cases. This likely results from the specific biology of the tumors in question. CRC, for example, is known to live at a “Goldilocks” state of balance when it comes to iron metabolism, in which both too much and too little may impair malignant behavior [21,56,57]. Indeed, CRC cases frequently have lower TFR1 expression than neighboring tissues due to iron overload and a need for protection from ferroptosis [23,58]. By contrast, cases driven by loss of the Adenomatous Polyposis Coli (*APC*) gene are associated with increased *TFRC* transcription and iron import [59]. Similarly, TNBC has a specific susceptibility to ferroptosis that likely diminishes the negative prognostic impact of high *TFRC* expression [60,61]. Indeed, drugs that increase TFR1 are toxic to these tumors for this reason [62]. Moreover, highly metastatic TNBC cells may specifically downregulate *TFRC*, which renders them sensitive to iron chelation [22]. Therefore, for both these tumor types, *TFRC*-high is likely driving a mix of both positive and negative biologic factors. In the case of CRC, there are likely to be differences by genetic subgroups that our dataset was unable to capture.

Recent advances in IO have illustrated a clear role of the immune system across solid tumors [1], and we sought to elucidate the relationship between *TFRC* and the tumor microenvironment across our selected cohort of solid tumors (BC, PRAD, NSCLC, and CRC). Prior reports have shown an association of *TFRC* or TFR1 with PD-L1, in single-tumor-type studies, some of which were restricted to IHC analysis [63,64,65]. We find significantly higher immune infiltration of lymphocytes and monocytes in *TFRC*-high tumors (Figure 3A). However, we also find significantly higher expression of genes encoding checkpoint proteins PD-L1, PD-1, LAG3, and CTLA-4, as well as immune-regulatory molecules, including CD80, CD86, HAVCR2, IDO1, and IFN-γ (Figure 2C). These data are associated with increased *TFRC* expression and TMEs that promote immune evasion by tumor cells. A natural way to target this is with immune checkpoint inhibitors. Our survival data show that while *TFRC* is prognostic in populations when ICIs are not used, this negative impact is reversed in NSCLC and is no longer significant in BC and PRAD cohorts. Transferrin receptor expression, indicating increased iron metabolism, should therefore be further examined as a potential positive biomarker for the use of IO. Although our results are derived from very large numbers of cases, the IO-treated and non-IO-treated groups were not necessarily matched for other clinical parameters. Validation should therefore be pursued in cases from randomized clinical trials with well-matched patient characteristics between groups. Moreover, our data defines transferrin receptor expression as a biomarker but not necessarily a driver of these effects. Immunocompetent in vivo models with titratable *TFRC* expression and subjected to detailed TME studies, including single-cell analyses, would be needed to test this relationship and establish mechanisms. Numerous downstream pathways are activated in association with TFR1 expression and iron import, including the JNK, ERK, HIF-1, and NFkB signaling cascades [20,66,67]. Several mechanistic routes could therefore connect to increased tumor PD-L1 expression and reprogramming of TMEs, and we have not investigated these possibilities here. Therefore, our results point to a biomarker that is targetable for numerous therapies under development as described above, while the mechanistic connections provide exciting avenues for future investigation.

A strong body of evidence has shown that immunotherapy’s favorable anti-tumor effects are at least partially facilitated by activating ferroptosis, a mechanism of iron-induced cell death [33,34]. In work by Chung et al. using samples from patients with head and neck cancer, ferroptosis is associated with inflammation, aggressiveness, and PD-L1 expression through activation of NF-kB. In a murine syngeneic HNSCC model, a synergistic effect of ferroptosis inducers with PD-L1 inhibitors was also observed [68]. Ferroptosis has also been reported to play a crucial role in the immune infiltration of clear cell renal cell carcinoma [69]. Moreover, TFR1 has been reported as a specific marker of ferroptosis [31], yet its biological role in ferroptosis in a clinically meaningful tumor context has yet to be elucidated. In analyzing *TFRC*-high tumors from our cohort, we find higher expression of *SLC7A11* and *SLC3A2* (system X_c_^−^ antiporter), well-studied genes that function to protect the cell against ferroptosis (Figure 4A). Our data does not establish a mechanistic link between TFR1 and expression of system Xc, but suggest these tumors require increased protection from ferroptosis and select for increased expression of the antiporter as they progress. Further exploring this finding using in vitro model systems, we find that iron chelation and iron supplementation lead to respective decreases and increases in expression of PD-L1 in TFR1-high lung cancer cell lines. We also show that iron supplementation promotes sensitivity to CD8+ T cell-mediated cellular killing, expanding on previous findings by others [34]. Our data therefore further supports a model in which iron utilization by tumors is associated with an increased requirement for immune evasion.

**Figure 5:**
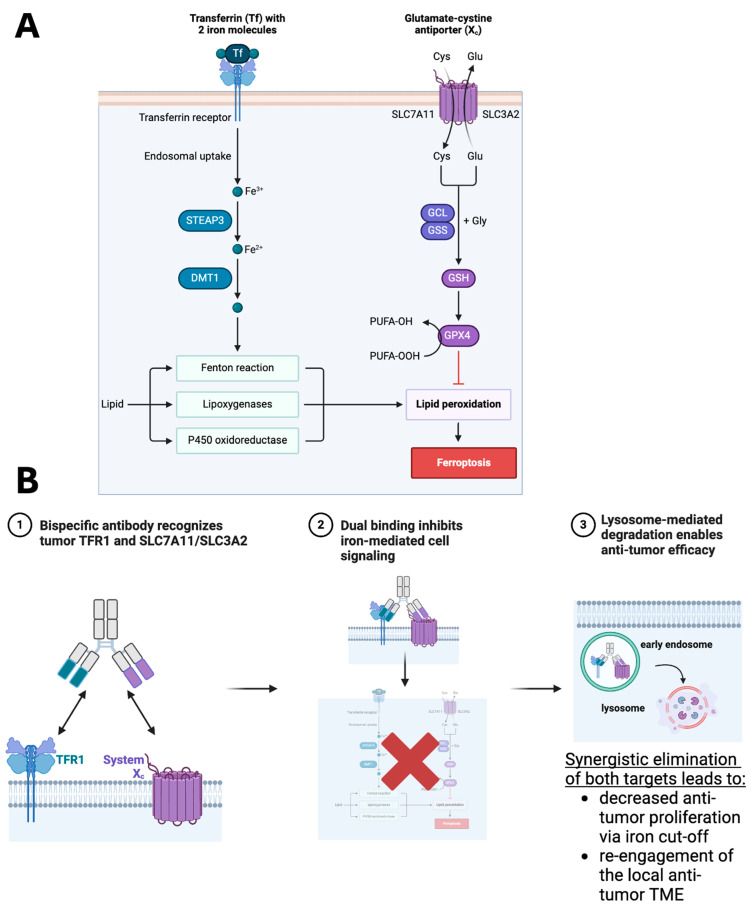
Working model for the therapeutic hypothesis of TFR1 and System X_c_^−^ as potential co-targets in cancer therapeutics. (**A**) Convergence and interaction of TFR1 and System X_c_^−^ (SLC7A11 and SLC3A2) on lipid peroxidation and death by ferroptosis. (**B**) Co-targeting strategies can leverage common co-over-expression of these targets in solid tumors (1), shutting down the pro-growth and protective signaling shown in (**A**) (2), through internalization and degradation (3).

Compounds targeting system X_c_^−^ led to the original discovery of ferroptosis [70]. Both SLC7A11 and SLC3A2 (CD98hc) have been reported to also play a role in promoting tumorigenesis [71,72], and indeed therapeutic strategies have been proposed against SLC3A2 [73]. One concern from monovalent drugging of either of these ferroptosis tumor targets with the recently proposed CAR-T [71,72,73] or ADC strategies is the potential for a resistance-mediated feedback loop, either through intracellular signaling or through tangential TFR1-mediated signaling (Figure 5A), as partially evidenced through our work here showing an intertwined biological relationship between these two protein classes. To maximize potential clinical benefit, we propose leveraging advances in Ab engineering technology [74], to develop cis-targeting bispecific degrader Ab co-targeting TFR1 and system Xc (SLC7A11/SLC3A2) (Figure 5B). These bispecific degraders would have a multi-modal mechanism of action that (1) antagonizes natural tumor proliferation-fueling iron-mediated signaling, (2) leads to lysosomal-mediated degradation of both tumor target proteins together, limiting potential for iron-signaling bypass and boosting anti-tumor efficacy driven by degradation over inhibition, and (3) re-engages the local immune system via activation of ferroptosis and re-activation of anti-tumor lymphocytes and monocytes.

Additional limitations inherent to the nature of our available data introduce caveats in the interpretation of our findings. Lack of access to detailed medical records, staging information, treatment response, treatment complications, and disease progression limits the analysis of survival data. We elected to study survival to IO agents to minimize prior therapy effects, but that also introduces uncertainty as to which line of therapy IO was used in; patients receiving IO in first-line versus in later lines can be expected to have different survival, and we were unable to adjust for that.

## 5. Conclusions

In conclusion, we provide robust evidence of the negative prognostic impact of *TFRC* expression in multiple common solid tumor types that are associated with an immune cell-infiltrated TME robustly expressing immune checkpoint molecules. We also provide a rationale for targeting these *TFRC*-high cancers with immune therapy that synergizes with targeting of ferroptosis and TFR1. Lastly, we propose a therapeutic strategy that synthesizes our collective findings into a polypharmacologic approach that could potentially deliver a synergistic therapeutic benefit.

## Data Availability

The deidentified sequencing data and corresponding clinical parameters analyzed in this report are owned by Caris Life Sciences. Qualified researchers can apply for access to these summarized data by signing a data usage agreement. Please request access at: https://www.carislifesciences.com/ (URL accessed on 25 April, 2026). Raw data from functional laboratory studies are available upon reasonable request from the corresponding author.

## Supplementary Materials

The following supporting information can be downloaded at: https://www.mdpi.com/article/10.3390/cancers18091402/s1, Figure S1: Pan-cancer prognostic analyses across molecularly stratified tumor types; Figure S2: *TFRC*-high and additional drivers.
