# Supplementary material for: Transferrin Receptor Overexpression in Solid Tumors Is Associated with Inflamed Microenvironments and Upregulated Immune Checkpoints, with Implications for Immunotherapy Sensitivity
Source: Cancers (Basel). 2026 Apr 28;18(9):1402. doi: 10.3390/cancers18091402 (PMC13163038; doi:10.3390/cancers18091402)
Supplement: Supplementary file 1 [file cancers-18-01402-s001.zip › cancers-4229771-supplementary/cancers-4229771 Supplementary/cancers-4229771 Western Blot Images.pdf]

Notes: The images were cropped to remove the protein ladders in main figures, shown here labeled for completeness. The left side of the image in 4B (included in the main figure) contains 20 ug of protein lysates from each of the indicated cell lines. The right side of the image contains the remainder volume from the samples used for the experiment. The right portion—containing the remaining sample volumes—was included inadvertently and does not provide additional experimental value. For all blots, the loading control-normalized relative densitometry (ImageJ v0.6.0) are included here as in the main figure for the experimental bands.

<u>Labeled images with edges</u>

Figure 4B:

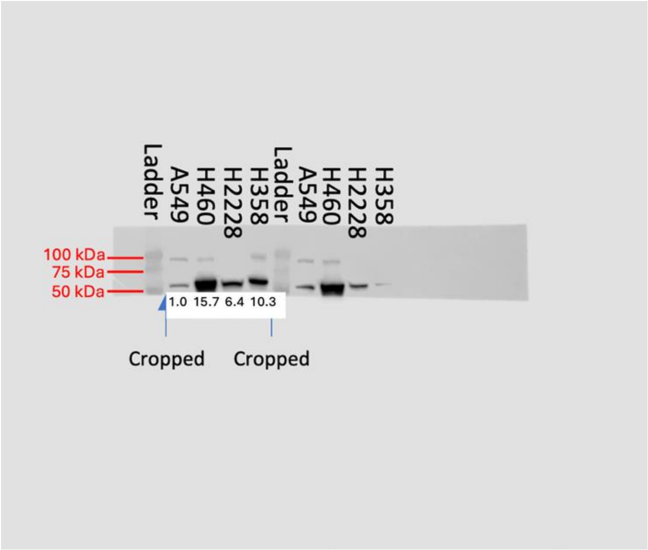

TFR1

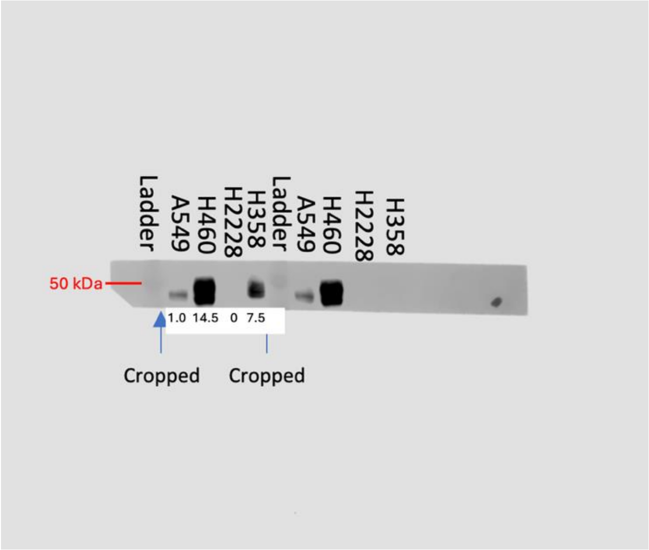

PDL1

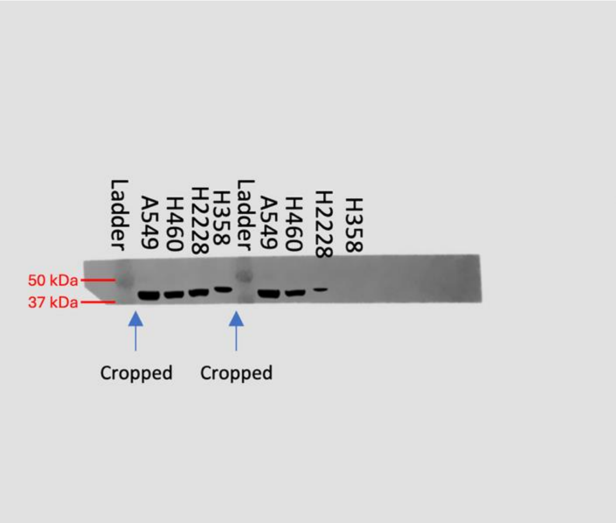

B-actin

Figure 4D
H460:

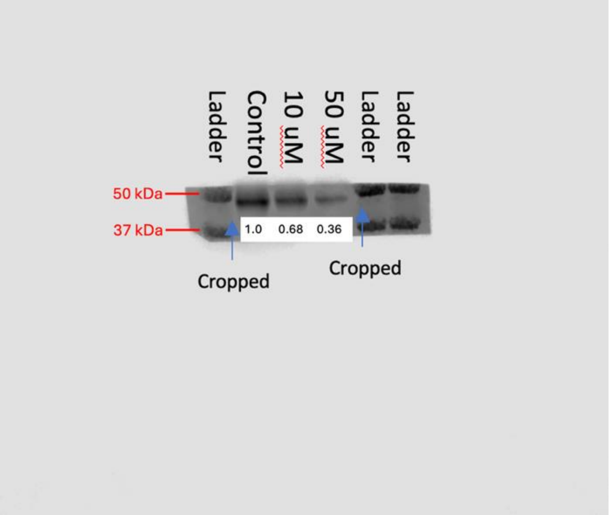

PDL1

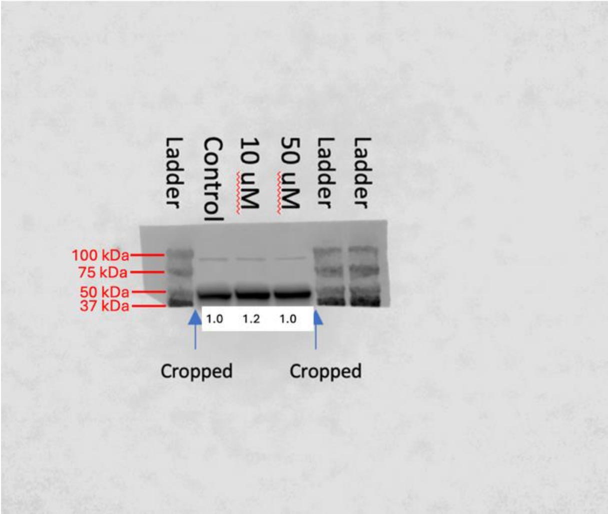

TFR1

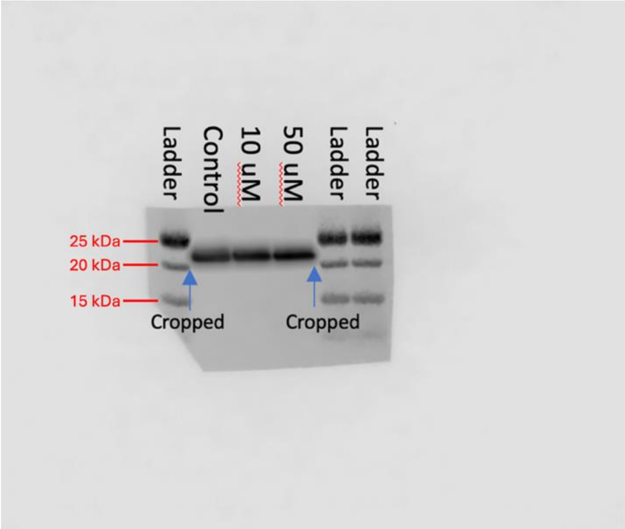

Cyclophilin

H358:

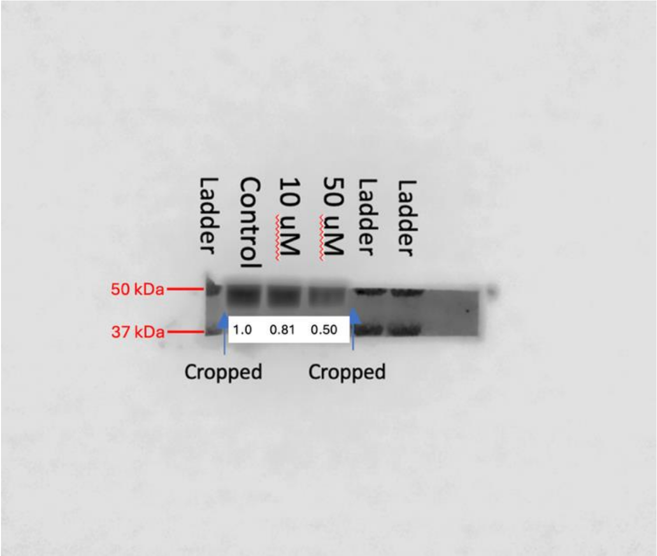

PDL1

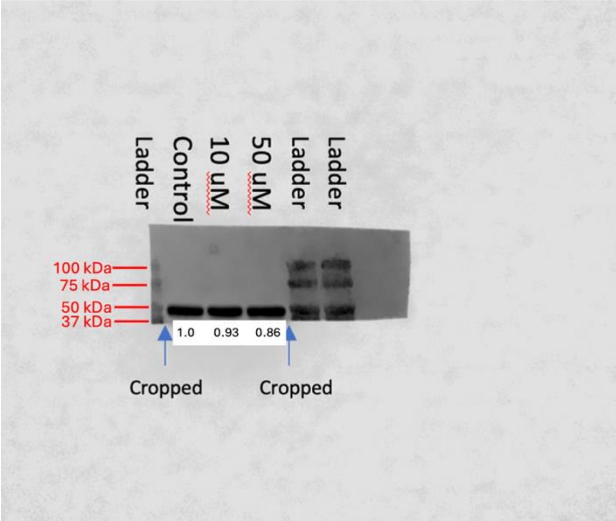

TFR1

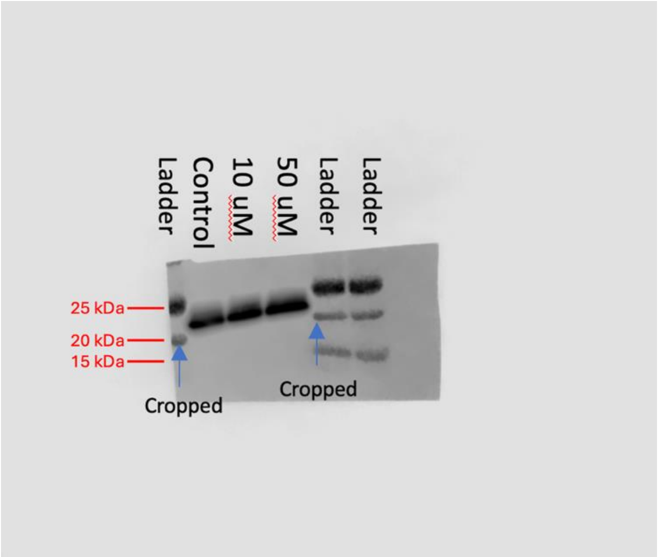

Cyclophilin

Figure 4E:

H460:

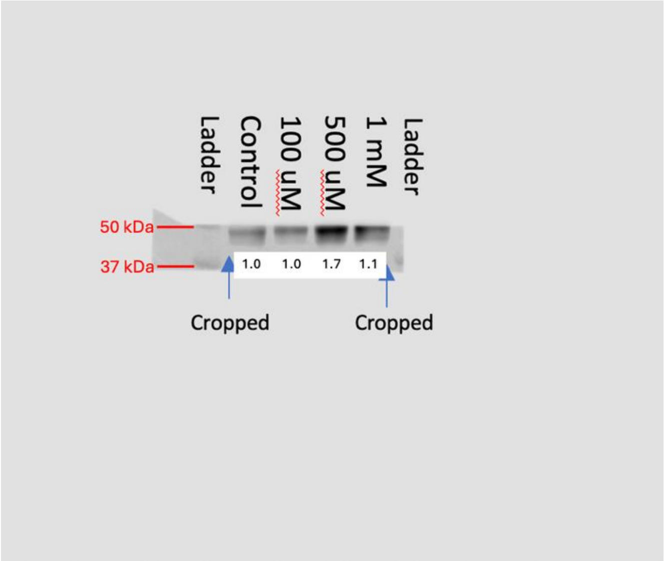

PDL1

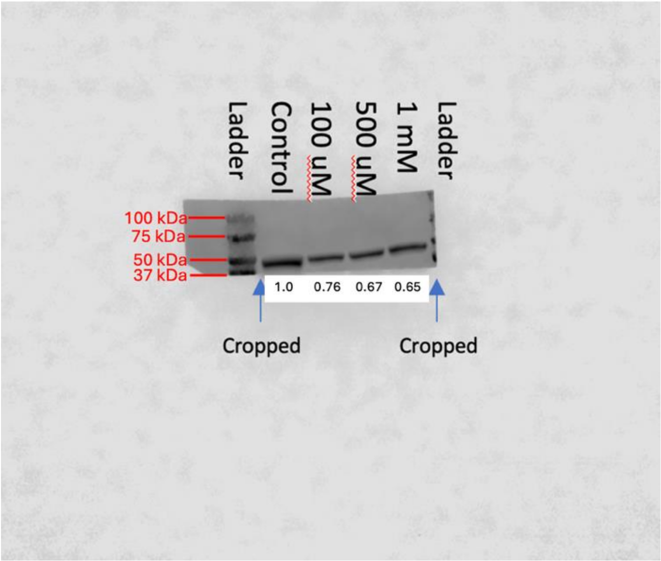

TFR1

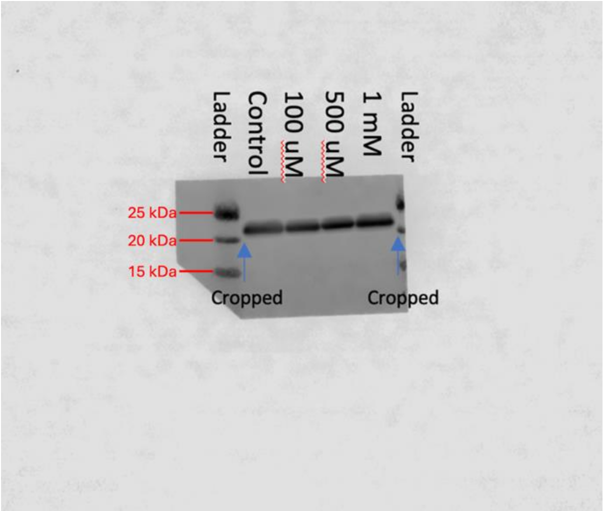

Cyclophilin

H358:

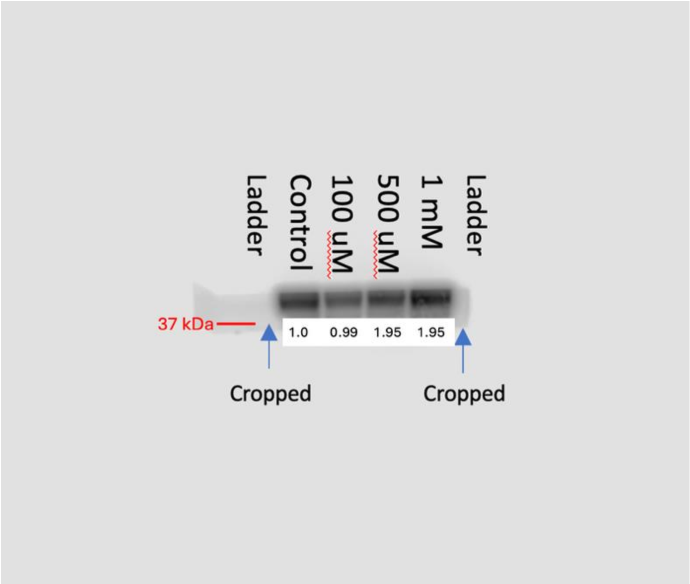

PDL1

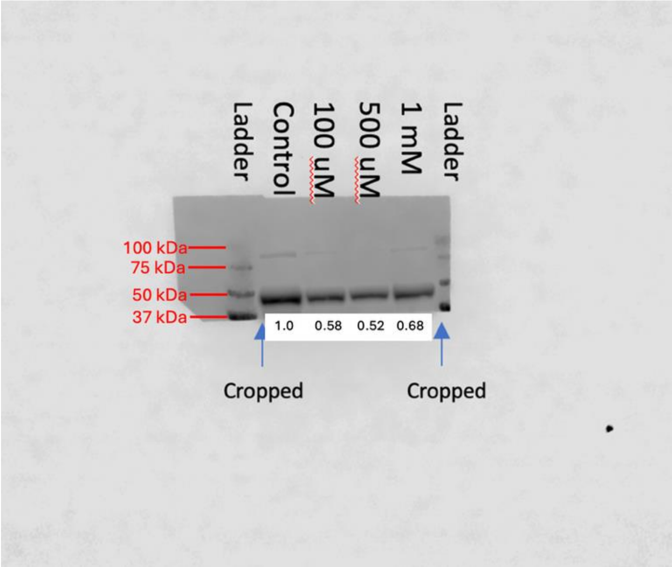

TFR1

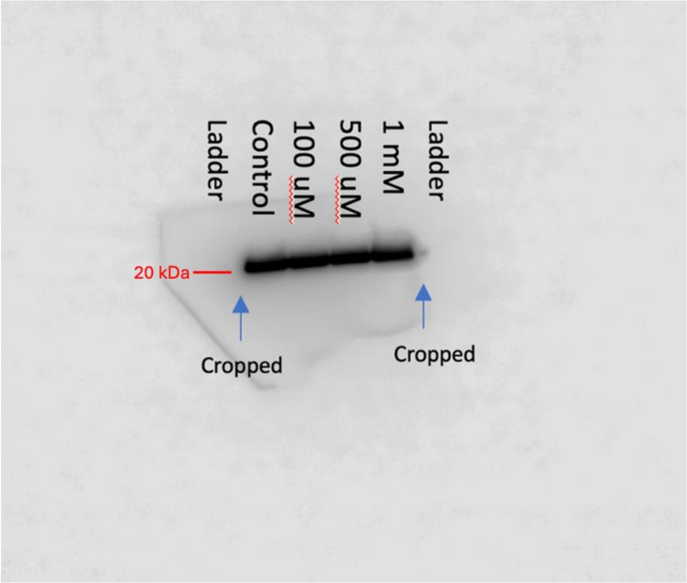

Cyclophilin
