# Supplementary material for: Transferrin Receptor Overexpression in Solid Tumors Is Associated with Inflamed Microenvironments and Upregulated Immune Checkpoints, with Implications for Immunotherapy Sensitivity
Source: Cancers (Basel). 2026 Apr 28;18(9):1402. doi: 10.3390/cancers18091402 (PMC13163038; doi:10.3390/cancers18091402)
Supplement: Supplementary file 1 [file cancers-18-01402-s001.zip › cancers-4229771-supplementary/cancers-4229771 Supplementary/cancers-4229771-supplementary.pdf]

Supplemental Figure 1A

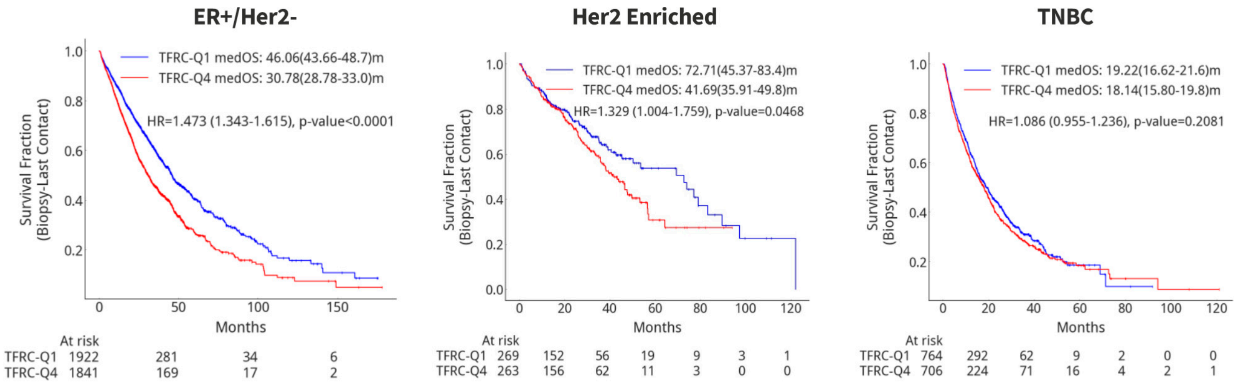

Supplemental Figure 1B

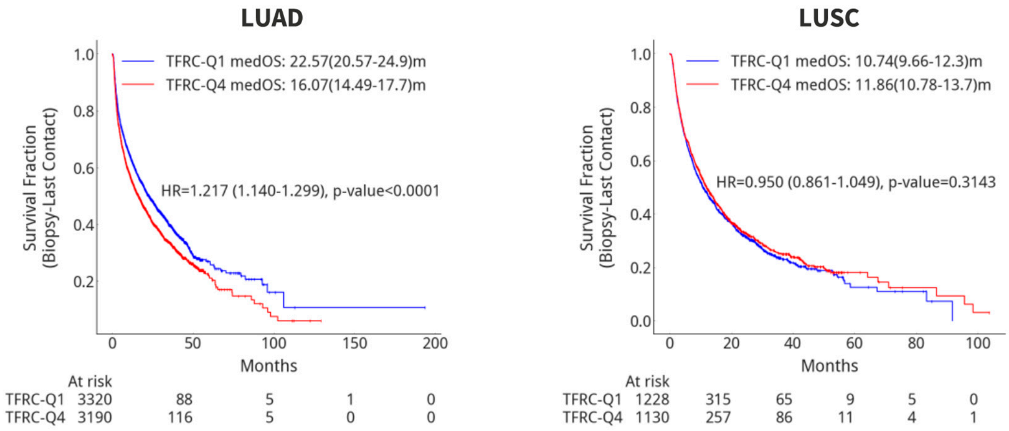

Supplemental Figure 1C

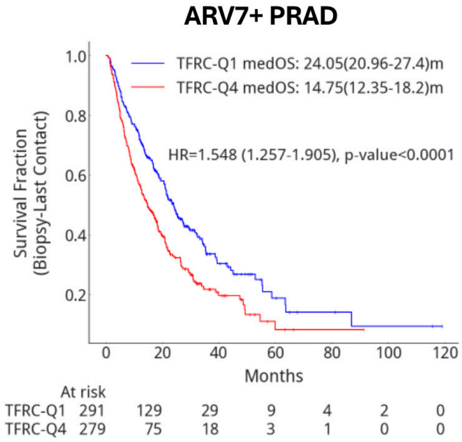

Supplemental Figure 1D

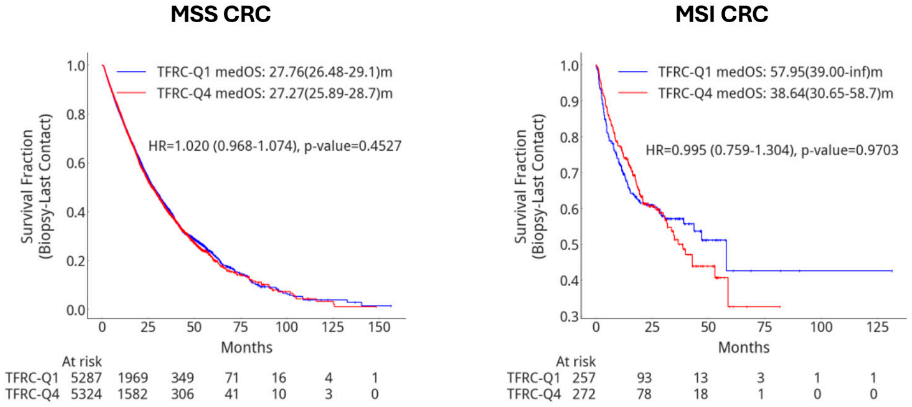

Supplemental Figure S1: pan-cancer prognostic analyses across molecularuly-stratified tumor types. Kaplan-Meier estimates for OS were analyzed in **(A**) estrogen receptor positive (ER+/HER2-), HER2 amplified, and TNBC, **(B)** lung adenocarcinoma (LUAD) and lung squamous cell carcinoma (LUSC), **(C)** androgen receptor splice variant 7-positive prostate cancer (AR-V7+ PRAD), **(D)** microsatellite stable (MSS) and microsatelitte instable (MSI) CRC. Cox proportional hazard model was used to estimate the HR for each tumor type with significance determined as *P* value of <0.05.

Supplemental Figure 2A

NSCLC				
covariate	HR	HR lower 95%	HR upper95%	p-value
TFRC-Q4	1.235813	1.167193	1.308467	3.75E-13
TP53	1.349861	1.26665	1.43854	2.44E-20
PIK3CA	0.96341	0.863173	1.075287	5.06E-01

Breast				
covariate	HR	HR lower 95%	HR upper95%	p-value
TFRC-Q4	1.234583	1.140112	1.336881	2.12E-07
TP53	1.642805	1.515265	1.781081	2.21E-33
PIK3CA	0.948012	0.878839	1.022629	1.67E-01

Prostate				
covariate	HR	HR lower 95%	HR upper95%	p-value
TFRC-Q4	1.770248	1.585013	1.977132	4.17E-24
TP53	1.571803	1.40769	1.755049	9.16E-16
PIK3CA	1.703796	1.35386	2.144181	5.55E-06

Supplemental Figure 2B

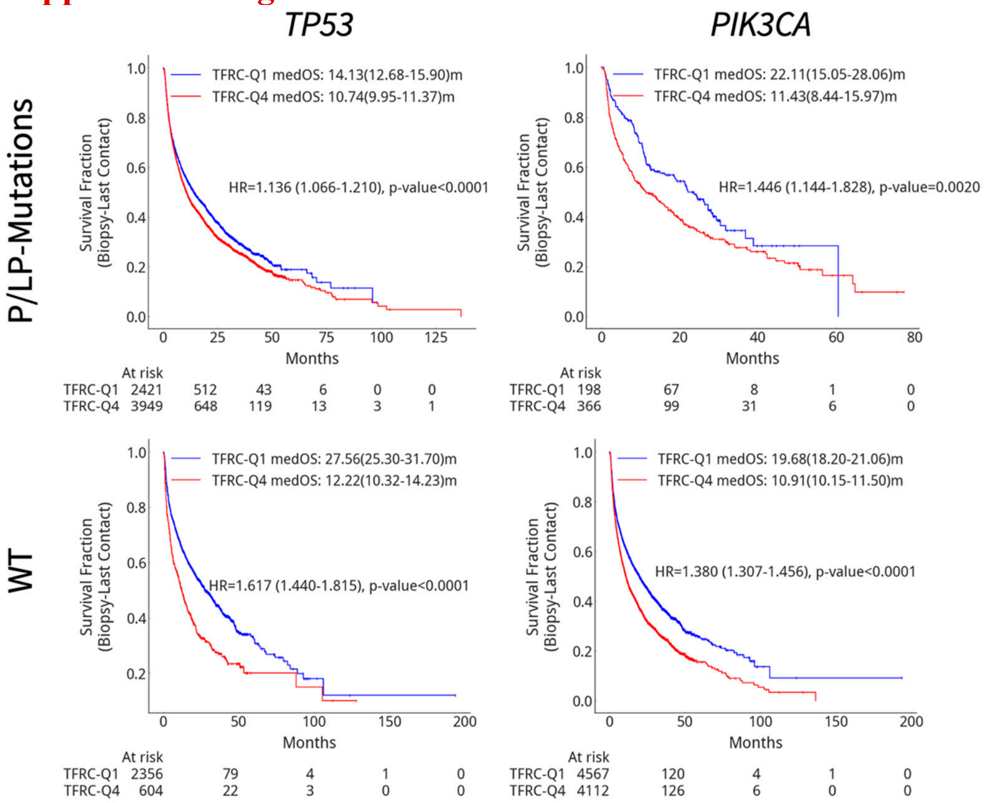

Supplemental Figure 2C

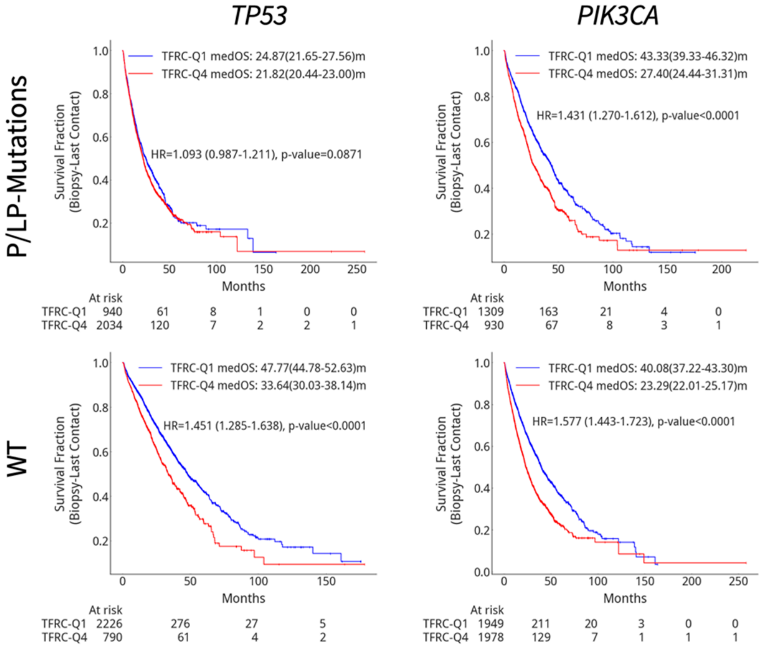

Supplemental Figure 2D

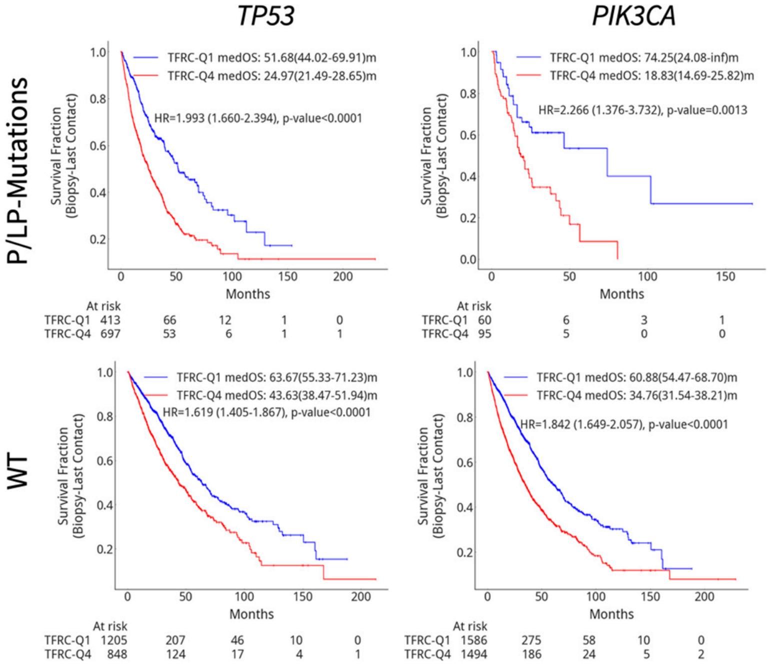

Supplemental Figure S2: *TFRC*-high and additional drivers. (A) Statistics tables for MVA results in **Fig. 2B. (B-D)** Kaplan-Meier estimates for OS assessing cases with and without (WT) *TP53* or *PIK3CA* mutations in lung (B), breast (C), and prostate (D) cancers. Cox proportional hazard model was used to estimate the HR for each tumor type with significance determined as *P* value of <0.05.
